# Identifying leisure constraints associated with acculturation among older Korean immigrants

**DOI:** 10.1080/17482631.2019.1655378

**Published:** 2019-08-27

**Authors:** Junhyoung Kim, Young Ik Suh, Jaehyun Kim

**Affiliations:** a School of Public Health, Indiana University, Bloomington, IN, USA; b Department of Sport Management, Wellness, and Physical Education, University of West Georgia, Georgia, GA, USA; c Department of Health and Human Performance, Texas State University, San Marcos, TX, USA

**Keywords:** Acculturation, leisure constraint, Older Korean immigrants

## Abstract

**Purpose**: Little research exists on understanding the interference factors that affect leisure engagement among older East Asian immigrants. Using the leisure constraint theory, this study was designed to identify leisure constraints associated with acculturation among older Korean immigrants.

**Method**: using semi-structured, in-depth interviews, a total of 18 individuals participated in this study.

**Results**: Three major themes were identified as leisure constraints related to acculturation: (a) adaptation challenges, (b) cultural norms, and (c) a lack of leisure opportunities. These identified themes served as inhibitors of the participants’ leisure involvement.

**Conclusion**: The findings suggested that acculturation-related experiences contributed to leisure constraints. Practical suggestions and implications are discussed in this study.

Participation in leisure contributes to physical, social, emotional, and cognitive wellbeing among older immigrants (Cuellar, Bastida, & Braccio, 2004; Khvorostianov, Elias, & Nimrod, 2012). Researchers suggest that leisure provided contexts in which older immigrants gained health benefits including psychological well-being, improved physical functions, and social benefits (Choi, Wilbur, Miller, Szalacha, & McAuley, 2008; Hernandez, 2012; Mock, Wilson, Smale, & Hilbrecht, 2013; Purath, Van Son, & Corbett, 2011). For example, prior studies have demonstrated that leisure helped older immigrants alleviate negative psychological symptoms such as feelings of loneliness and depression and foster positive social interactions with others (Hamer, Molloy, de Oliveira, & Demakakos, 2009; Kim, Chun, Heo, Lee, & Han, 2014; Lampinen, Heikkinen, & Ruoppila, 2000).

Multiple studies have stressed the importance of understanding the interference factors that affect leisure participation among older adults as they inhibited leisure choices and leisure satisfaction (Chick, Hsu, Yeh, & Hsieh, 2015; Spiers & Walker, 2009). From the perspectives on acculturation, older immigrants encounter various challenges such as language barriers, limited social networks, and cultural differences (Hsu, Davies, & Hansen, 2004; Hwang & Ting, 2008). Such adaptation challenges may inhibit older adults’ leisure participation that results in diminished life satisfaction.

Jackson (1988) defined such inhibiting factors of leisure engagement as leisure constraints. These factors “inhibit people’s ability to participate in leisure activities, to spend more time doing so, to take advantage of leisure services, or to achieve a desired level of satisfaction” (Jackson, 1988, p. 203). The leisure constraint theory implies that constraints may reduce or alleviate participation or satisfaction in leisure activities. It is important for leisure service providers and recreational therapists to understand how the nature and intensity of constraints affect leisure behaviours of older immigrants (Kazeminia, Chiappa, & Jafari, 2015; Palen et al., 2010).

Golob (2010) suggested that leisure service providers and recreational therapists need to minimize constraint factors of leisure engagement among older adults who have diverse cultural and ethnic backgrounds. The background of this suggestion is that older immigrants can have unique constraint factors related to acculturation that negatively affect leisure choices and leisure engagement. Thus, it is imperative that leisure service providers and recreational therapists exert significant efforts to minimize leisure constraints, which can facilitate leisure participation among older immigrants. It also can contribute to their quality of life and their adaptation process into mainstream society.

In spite of the importance of leisure behaviours associated with leisure constraints, little research exists that explores what factors inhibit leisure engagement among older immigrants. By exploring leisure constraints that older immigrants experience, leisure service providers and recreational therapists can design and implement effective programs and activities that can promote health and well-being.

For this study, older Korean immigrants were selected as a sample for several reasons. First, the number of older Korean immigrants is continuously increasing and, currently, constitutes the fourth largest Asian immigrant group in the U.S. (Berkman & Ko, 2009). Second, older Korean immigrants reported experiencing a lower level of acculturation and more language barriers that other East Asian immigrants (Jang & Chiriboga, 2011; Sung, 2001). Third, exploring health-related issues and acculturation needs to be expanded in order to increase health promotion among older Korean immigrants. Therefore, the purpose of this study was to identify the leisure constraints associated with acculturation among older Korean immigrants. This study approached the meaning of leisure as a fun, entertaining activity that older Korean immigrants engage in during their free time.

## Leisure constraint theory and immigrants

Leisure scholars have developed a variety of conceptual and methodological models to help explain how leisure constraints may operate (Caldwell & Baldwin, 2005; Chick et al., 2015; Hudson, Walker, Simpson, & Hinch, 2013; Kyle & Jun, 2015). According to Jackson, Crawford, and Godbey (1993), three main components exist that restrict an individual’s leisure participation: (a) intrapersonal, (b) interpersonal, and (c) structural. Intrapersonal constraints are defined as psychological attributes that affect the development of leisure preferences, such as a lack of leisure interests and skills, negative psychological symptoms, low self-esteem and confidence, and limited socialization into specific leisure activities (Caldwell & Baldwin, 2005; Crawford & Godbey, 1987). Interpersonal constraints reflect interpersonal interactions, including an inability to discover leisure partners (Chick et al., 2015; Crawford & Godbey, 1987). Structural constraints are external factors that intervene between leisure preferences and participation, such as problems related to limited facilities, service accessibility issues, and lack of finances (Dhurup, 2012; Kazeminia et al., 2015). Based on the leisure constraints theory, ethnic and cultural differences are examples of structural constraints.

Stodolska and Yi-Kook (2005) focused on the theoretical constructs associated with constraints on the leisure of immigrants. They identified three important constraining factors related to immigrants’ leisure constraints: (a) monetary and time constraints, (b) lack of English skills, and (c) lack of established social networks. They also suggested that immigrants are likely to experience more constraints than other ethnic groups because both immigration-related constraints and general leisure constraints influence their participation in leisure activities. The current study incorporates this theoretical model into understanding leisure constraints of older Korean immigrants.

According to Yu and Berryman (1996), Chinese adolescent immigrants identified linguistic barriers as the main leisure constraints. These challenges prevented them from seeking leisure partners and utilizing leisure resources. They also found that leisure constraints that they experienced were negatively associated with levels of self-esteem. Juniu (2000, 2002)), who studied South American immigrants, reported that deficient communication skills and cultural and ethnic differences served as leisure constraints. Ethnic and cultural differences have also been identified as constraints detrimental to the leisure pursuits of immigrants. For example, Livengood and Stodolska (2004) explored the effects of ethnic and cultural differences on the leisure behaviours of American Muslims. They found that the participants modified leisure behaviours because of differences in culture and ethnicity.

Some researchers have demonstrated that acculturation, gender, and culture are associated with immigrants’ leisure constraints. For example, Tsai (2000) explored the influence of acculturation on the perception of six leisure constraints (i.e., socio-cultural constraints, interpersonal constraints, access constraints, affective constraints, physiological constraints, and resources constraints) in a study of 127 Chinese immigrants. In this study, the participants, who were more acculturated, reported that they had lower levels of socio-cultural, interpersonal, and access constraints. Tcha and Lobo (2003), who studied Korean immigrants in Australia, found that female immigrants were more likely to perceive leisure constraints to be binding than males and, as such, reported less acculturation. In addition, they found that female immigrants were less exposed to interactions with others of different cultural and ethnic backgrounds. Furthermore, acculturated individuals perceive fewer leisure constraints as they have increased access to leisure settings (Afable-Munsuz, Ponce, Rodriguez, & Perez-Stable, 2010).

Walker, Jackson, and Deng (2007) focused on three components of leisure constraints (i.e., intrapersonal, interpersonal, and structural) when performing a cross-cultural comparison between Canadian and Chinese university students. They found that the Chinese students perceived more interpersonal constraints, while the Canadian students perceived more structural constraints. Hence, both cultures appear to affect the different components of leisure constraints.

## Methods

### Research design

This qualitative study adopted semi-structured in-depth interviews to capture leisure constraints associated with acculturation. This method can allow researchers to explore and interpret participants’ personal life journeys in a detailed and vivid manner (Crabtree & Miller, 1999; Hesse-Biber & Leavy, 2005). Through the in-depth interviews, the authors obtained an in-depth of understandings and information related to leisure and acculturation and identified factors that restricted leisure participation.

### Participants and data collection

This study used a purposeful criterion sampling strategy suggested by Patton (2002). To satisfy the criteria, the participants immigrated from South Korea, were over 65-years-old, and understood either English or Korean. The authors recruited the study participants from various locations, including Korean senior centres, Korean communities, and Korean-based churches. In order to facilitate recruitment, the authors adopted a snowball sampling technique suggested by Freeman, Palmer, and Baker (2006). To identify and protect the participants, only a pseudonym was used. The University Institutional Review Board approved these procedures.

A total of 18 individuals participated in this study. Ten of the participants were males and eight were females. Their ages ranged from 65 to 85 (M = 72.2). The average length of time since their immigration was 34 years .The sample size was determined based on the theoretical saturation point where no new data emerged (Mack, Woodsong, MacQueen, Guest, & Namey, 2005).

Based on the literature review, the authors conducted semi-structured, in-depth interviews in order to examine what constraints older Korean immigrants experienced related to their leisure participation. Each interview lasted between 50 and 90 minutes. The interview locations were selected by the participants and included senior centres, cafeterias, and homes. As preferred by the participants, the authors conducted all of the interviews in Korean. This study employed general and specific questions in order to capture the leisure constraints experienced by the participants. Examples of the general questions are “Do you feel most comfortable speaking in English or Korean?” and “Could you tell me about your life story?” Examples of the specific questions are “Please tell me about the kinds of things you do for fun or enjoyment in your free time” and “Do you experience any challenges when seeking to participate or participating in these activities? If yes, please tell me what it is.” All of the interviews were audio-recorded and transcribed verbatim. After conducting the interviews, the participants were asked to respond to demographic-related questions.

### Data analysis

This study incorporated the guidelines for data analysis suggested by Creswell (2009). First, we produced the raw data by making transcriptions of each participant’s interview. For the data analysis, we organized and prepared each data set and read it thoroughly. While reading and re-reading each transcript, we created a list of general ideas, labelled “natural meaning units.” Across each transcript, general themes and subthemes were determined and interpreted. Using the constant comparative method (Merriam, 1998), we simultaneously analysed one data from others.

### Trustworthiness in qualitative research

Qualitative researchers have adopted different techniques to increase the credibility of their qualitative data (Creswell, 1998; Kvale, 1996). Among these techniques, two techniques were chosen to be used in this study. First, member checking was performed to verify the data interpretations with the participants by determining their satisfaction with the data interpretations as suggested by Peterson, Sword, Charles, and DiCenso (2007). In addition, in order to increase the accuracy of the transcripts, the authors followed the guidelines suggested by Suh, Kagan, and Strumpf (2009). Several paragraphs were randomly selected and translated from Korean to English by bilingual research scholars. After their translation, the authors held a conference call to validate the quality and conceptual equivalence of the translation. They agreed that the conceptual meanings and contents of the translation did not include any concerns.

## Findings

After the data analysis, significant differences were obvious in gender and acculturation related to leisure constraints. For example, 14 of the participants experienced challenges associated with leisure participation related to their culture and immigration experiences. Four of the participants mentioned that they were well-adjusted in their host culture and did not describe any challenges associated with leisure participation. Based on the above information, three major themes were identified as leisure constraints related to acculturation: (a) adaptation challenges, (b) cultural norms, and (c) a lack of leisure opportunities. These constraints served as inhibitor of the participants’ leisure involvement.

### Adaptation challenges

Experiencing adaptation challenges was the most salient theme that restricted the participants’ leisure engagement. Among various types of adaptation challenges, language barrier was the most restricting factor that influenced leisure participation such as a lack of motivation to use recreational facilities. For example, they mentioned that they experienced communication issues with leisure service providers because of language barriers. For example, Lee-F (female, 75) mentioned that she had a difficult time collecting information on the locations and schedules of recreational programs and events.

… I walked on the track and some people explained to me the track rules many times. I did not understand what they told me … One day, my daughter explained to me that there were three different lanes that were for walking, jogging, and running. I realized that I had interrupted so many people because I always walked on the running lane.

She said that she was reluctant to maintain her membership at the facility because she felt embarrassed.

Such challenges discouraged them from participating in activities as members of the recreational facilities. In particular, the female participants pursued sedentary leisure behaviours because of their deficient language skills. Even when they participated in programs at senior centres, they experienced language barriers and had feelings of loneliness and isolation in group recreational settings. They preferred to find the Korean senior centres where they could interact with other Korean immigrants. For example, Kwon (female, 70) stated,

It was very inconvenient to live here because I did not speak English well … but I felt bored because the only thing I was supposed to do was to take care of my children at home. That’s all I could do as a grandmother.

Limited social networks and language barriers served as factors that limited her socialization with others.

A new life and cultural environment restricted some of the participants to pursue leisure engagement. It appears that a different way of lifestyle negatively affected leisure participation such as the use of transportation. For example, some of the female participants mentioned that they could easily access leisure facilities, such as parks, senior centres, and gyms using public transportation in Korea, but, after their immigration, they had difficulty time in utilizing the public transportations because of a lack of transit options. As such, they were dependent on their adult children for their transportation. They mentioned that without their children’s help, they could not go anywhere to socialize with others and engage in leisure activities.

Such adaptation difficulties prohibited them from participating in various activities and served as constraints to leisure participation. For example, Lee (female, 77) and Chen (female, 66) could not drive and, therefore, they could not really engage in any activities. They mentioned that even though they intended to participate in social events, their inability to drive restricted them from participating. They also stated that they were unable to go to public parks to get fresh air or relieve their stress because of their inability to drive. They felt embarrassed asking their children for transportation because they were busy with work.

Jo (female, 70) said that, since her immigration, she was expected to take care of the household chores as well as help at her husband’s business, which, in turn, caused her to have a lack of leisure time.

[America] is a fast-paced society and I did not have much leisure time for myself. In this American life, I should work and make money because we could not survive with only my husband’s financial support. In Korea, there were many women who did not work … I am supposed to work so hard and do not have any personal time for myself. It was so difficult to live and do something for myself.

While she felt satisfaction helping with her family’s financial situation, her obligations left her with little time for leisure pursuits.

These examples indicate that the participants experienced adaptation challenges, such as differences in languages, lifestyles, and culture. Such challenges served as leisure constraints that restricted leisure participation among the participants

### Cultural norms

Fifteen of the participants mentioned that they had a moral obligation and expectation to live with their adult children and grandchildren. Even though three of the participants lived independently, they also believed that taking care of their grandchildren was their top priority. Due to this priority, they said that they did not have any personal time within which to enjoy their leisure time. For example, Kwon (female, 70) said that caring for her grandchildren was like a full-time job. By spending most of their free time with their grandchildren, the participants gained some benefits, such as positive intergenerational relations.

In spite of some benefits of taking care of grandchildren, they mentioned that they sacrificed their personal time and energy to taking care of their grandchildren. This cultural norm as grandparents prevented them from pursuing other meaningful activities on their own. For example, Lee-F (male, 85) said,

It was fun to play with my grandchildren. However, I had to give up playing golf with my friends during the day because of this. Sometimes, I wanted to say to my son that I needed a break, but he is my son and need our help.

He also mentioned that, sometimes, he was physically and mentally exhausted from taking care of his grandchildren and felt that he did not have any energy left for other activities, which further inhibited him from engaging in leisure activities.

Not only most of the female participants experienced a lack of personal leisure time by taking care of grandchildren, but did they household tasks, which prevented them from participating in leisure activities. They mentioned that Korean culture demanded that daughters-in-law look after their mothers-in-law when they became weak and sick. The cultural belief system indicates that daughters-in-law should prepare the meals and take care of the household chores. However, since immigrating, these older women have encountered the opposite situation. For example, Lee (female, 77) stated,

When I lived in Korea, my family supported and respected me as an older adult. They provided me with many things and my life was not difficult. After my immigration, when my son and daughter-in-law went to work, all of the household chores have become my responsibility. Taking care of the household chores keeps me busy and I have had no chance to feel lonely or do something for myself.

This cultural role transition created stressful and distressing situations for her.

Only three of the male participants mentioned that they experienced some challenges to participating in leisure activities. For example, Lee-W (male, 65) maintained a strong sense of his gender role in that he only focused on his responsibility of taking care of his family. Korean culture demanded that males should be responsible for their families and expects that their wives should stay at home to take care of the children. This acculturation pressure enabled him to engage in unhealthy behaviour, which led to issues with alcohol. Due to this unhealthy behaviour, he mentioned that he could not strive to do something meaningful or enjoyable for his personal life.

I drink every day. Whenever I felt bored and lonely, I drink a lot. I did not know what I could do for myself except work. It is a miracle that I am still alive here … I could not enjoy doing something for fun because of many challenges.

He also mentioned that he did not utilize any resources related to leisure because of his work and alcohol issues. It appears that adaptation difficulties can be negatively associated with negative leisure behaviours and attitudes.

Based on the participants’ experiences and statements, it appears that the participants maintained their cultural values and beliefs and embraced new cultural norms at home that served as leisure constraints. Taking care of grandchildren was the main contribution to leisure constraints. In addition, a gender difference existed in regard to leisure constraints. It seems that the female participants perceived more adaptation challenges, such as cultural differences and new cultural roles, that results in constraints to leisure participant than the male participants. Only three of the male participants experienced leisure constraints due to a cultural gender role difference.

### Limited leisure opportunities

All of the participants mentioned that they had limited opportunities to utilize a variety of leisure resources and leisure opportunities. They identified Korean senior centres and Korean churches as major avenues of leisure programs. While they expressed an appreciation for and the value of their engagement in programs offered by these organizations, they experienced challenges related to expanding their leisure choices and leisure pursuits. Lee-W (male, 65) said that he had a difficult time in exploring new leisure activities because of a lack of information related to such activities. For example, Park (male, 73) stated,

I was so familiar with the programs and activities offered by the Korean senior centers. Sometimes, I desired to meet new people and do activities with others whom I was not familiar with. I did not mean that I did not like these programs. I did not know where to go for other activities and to meet new people.

He expressed an interest in expanding his social networks through his leisure participation.

Some of the participants tended to pursue the same leisure activities that they had participated in while living in South Korea. Due to a different cultural setting, they had experienced challenges related to maintaining their preferred activities in the host country. For example, a few of the participants had been enthusiastic gateball players in Korea and had limited opportunities and resources to play gateball. Sujin (female, 68) said that she failed to continue playing gateball since her immigration because she could not find any facilities that could accommodate this activity. In addition, Lee-W (male, 65), who played badminton, sought to evaluate his skills by playing other players. Even though he enjoyed playing badminton games with other older Korean adults, he mentioned that he desired to play the games with other advanced players. He shared that he experienced a lack of sports partners with different skillsets.

These participants’ experiences indicate that they had limited opportunities to access leisure facilities and maintain their preferred activities due to a lack of resources. They also indicate that a lack of leisure resources and limited opportunities serve as inhibitors of leisure participation.

## Discussion

This study attempted to identify leisure constraints associated with acculturation among older Korean immigrants. It suggests that culture-related factors serve as inhibitors of leisure participation. The findings of this study support previous findings that adaptation challenges such as language barriers and cultural and ethnic differences prevented immigrants from pursuing a variety of leisure activities (e.g., Juniu, 2000, 2002; Livengood & Stodolska, 2004; Rublee & Shaw, 1991; Yu & Berryman, 1996). With these leisure constraints, the current study extends the body of knowledge affirming that adaptation difficulties serve as constraining factors that restrict older Korean immigrants’ leisure participation. The results demonstrate that language barriers, transportation issues, limited social networks, and cultural and ethnic differences are all constraining factors. Thus, this study suggests that various constraining factors may be associated with participants’ leisure involvement.

Prior studies have suggested that gender is a mitigating factor in leisure, recreation, and sports participation for immigrants (Marquez & McAuley, 2006; Tcha & Lobo, 2003). Based on these findings, this study suggests that female participants have more constraints to leisure because of cultural differences and their dependence on adult children. This finding supports Tcha and Lobo (2003) study, which found that female immigrants perceive more leisure constraints than their male counterparts. From a cultural perspective, reflecting Confucian values, daughters-in-law support their mothers-in-law in various ways, such as providing meals and doing household chores. After immigration, this cultural value becomes reversed, which causes cultural conflicts between older female participants and their daughters-in-law. Such cultural conflicts are related to leisure constraints because they result in a lack of time that can be used to engage in meaningful activities for oneself.

The findings of this study suggest that age differences exist in leisure constraints among immigrants. Prior studies have found that adult immigrants identified a lack of time as their major leisure constraints (Scott, Lee, Lee, & Kim, 2006; Stodolska & Yi-Kook, 2005). The current study found that a lack of time was not significantly considered to be a leisure constraint even though older immigrants may have more leisure time. Cultural obligations to take care of and live with adult children and grandchildren were the main contributing factors in the participants’ lack of time, which caused leisure constraints.

Previous findings have suggested that adaptation difficulties, such as language barriers and cultural and ethnic differences, prevented immigrants from pursuing a variety of leisure activities (e.g., Juniu, 2000, 2002; Livengood & Stodolska, 2004; Rublee & Shaw, 1991; Yu & Berryman, 1996). With these leisure constraints, this study extended the body of knowledge to affirm that adaptation difficulties serve as constraining factors that restrict older Korean immigrants’ leisure participation. The results demonstrate that language barriers, transportation issues, limited leisure opportunities, and cultural and ethnic differences are constraining factors. Thus, this study suggests that various constraining factors may be associated with the participants’ leisure involvement.

### Limitations and future studies

Some of the limitations of this study should be addressed. First, this study mainly focused on leisure constraints of older Korean immigrants. Older immigrants from different countries may identify different factors that negatively affect their leisure engagement. If future researchers investigate leisure constraints of older immigrants from different countries, they can produce more valuable insights and information. In addition, the current study did not capture other factors that can influence leisure constraints such as a level of acculturation and demographic information. Future studies are needed to investigate what other factors are associated with leisure constraints among older immigrants. Last, different age groups can perceive leisure constraints in a different manner. It would be interesting if future researchers compare and contrast the relationship between young immigrants and older ones in terms of leisure constraints.

## Implications and conclusion

Multiple strategies exist that leisure service providers and recreational therapists can use to minimize leisure constraints experienced by older Korean immigrants and, as a result, facilitate leisure engagement. One strategy is to educate leisure service providers on how to communicate with older immigrant participants. By providing older immigrants with brochures in multiple languages, older immigrants can access various programs and resources offered at recreational facilities. In addition, it would be important to design and implement culture-related activities and programs with which older immigrants are familiar, such traditional games and activities. By creating recreational activities originating in their own countries, they can expand leisure choices and utilize leisure resources.

Leisure service agencies and organizations need to provide training programs for their staff members. Through multicultural training programs, leisure service providers and recreational therapists can develop a sense of cultural competence by gaining cultural knowledge and building confidence in working with older immigrants. In addition, they need to provide older immigrants with bilingual facility and program information so that they can utilize the facilities in a more effective manner.

This study identified the leisure constraints experienced by older Korean immigrants and provided practical suggestions on how to minimize them and facilitate leisure engagement. By understanding leisure constraints, leisure service providers and recreational therapists can provide the most appropriate programs and activities for their clients from different countries.
